# Commentary: SU9516 increases α7β1 Integrin and Ameliorates Disease Progression in the mdx Mouse Model of Duchenne Muscular Dystrophy

**Published:** 2017-08-29

**Authors:** Apurva Sarathy, Andreia M. Nunes, Tatiana M. Fontelonga, Tracy Y. Ogata, Dean J. Burkin

**Affiliations:** 1 Department of Pharmacology, University of Nevada, Reno School of Medicine, Reno, NV 89557, USA; 2 Departamento de Biologia Animal, Centro de Ecologia, Evolução e Alterações Ambientais, Faculdade de Ciências, Universidade de Lisboa, 1749-016 Lisbon, Portugal

## Duchenne muscular dystrophy

Duchenne muscular dystrophy (DMD) is a debilitating X-linked neuromuscular disease with an incidence of 1 in every 5000 boys^1^. It is caused by mutations in the *DMD* gene coding for dystrophin, a critical structural protein in muscle. Out-of-frame mutations in the *DMD* gene, result in the complete loss of dystrophin in muscle fibers^2,3^ and this leads to a severe disease characterized by progressive muscle deterioration. The functional role of dystrophin is to stabilize the dystrophin glycoprotein complex (DGC), which is composed of sarcolemmal glycoproteins that link the extracellular matrix (ECM) to the actin cystoskeleton in muscle fibers^4,5^. In the absence of dystrophin, this critical link is lost, rendering the muscle fibers susceptible to damage and contraction-induced injury. Until recently, palliative interventions such as glucocorticoid and corticosteroids were the only options available for disease management in DMD patients. These were accompanied by numerous side effects including weight gain, stunted growth, cataracts and susceptibility to skeletal fractures^6,7^. In September 2016, the Food and Drug Administration approved a drug for the treatment of patients with amenable mutations in exon 51 of the dystrophin gene. The drug Eteplirsen, a phosphorodiamidate oligonucleotide (PMO), is an exon skipping molecule^8^ which skips its target exon 51, thereby restoring the dystrophin translational reading frame and enabling expression of a truncated dystrophin molecule in patient muscle fibers. Eteplirsen addresses only 13% of DMD patients because it is mutation-specific^9^ and a therapeutic that can be universally administered to all DMD patients is still needed.

The α7β1 integrin is also a transmembrane protein in myofibers that links laminin in the ECM to the actin cytoskeleton, and studies have shown it can be harnessed as a compensatory system for dystrophin loss in DMD. The α7β1 integrin is the predominant laminin-binding integrin in skeletal, cardiac and vascular smooth muscle^10^ where it plays a structural role and participates in inside-out and outside-in cell signaling mechanisms that contribute to muscle development and physiology^11^. Loss of the α7 integrin in dystrophin deficient *mdx* mice exacerbates the dystrophic phenotype and mice do not survive past 4 weeks of age^12^. Conversely, transgenic overexpression of α7β1 integrin ameliorates disease pathology and improves survival in severely dystrophic mice^13^. Mechanisms that contribute to α7 integrin-mediated rescue of dystrophin-deficient muscle include maintenance of myotendinous and neuromuscular junctions, enhanced muscle hypertrophy and regeneration, and decreased apoptosis and cardiomyopathy^12–16^. Recent evidence suggests that prednisone may maintain function in the golden retriever muscular dystrophy (GRMD) dog model of DMD by stabilizing α7 integrin protein levels^17^. Together, these observations support the idea that the α7β1 integrin is a major disease modifier in DMD, and a target for drug based therapeutics. This led us to undertake a study to identify integrin enhancing small molecule compounds as potential treatments for DMD. In collaboration with a team of researchers at National Center for Advancing Translational Sciences (NCATS), our lab performed high throughput drug screens on several chemical libraries and screened over 350,000 compounds utilizing a muscle cell-based assay. Among the top hits identified in this assay was SU9516, a small molecule compound that increased α7 integrin levels in myoblasts and myotubes by >2-fold. In the original manuscript titled “SU9516 increases α7β1 Integrin and ameliorates disease progression in the *mdx* model of Duchenne muscular dystrophy”,^18^ our research group showed that a small molecule compound, SU9516, significantly increases muscle function and improves pathology in the *mdx* mouse model of DMD. Additionally, we found that these improvements were at least partially mediated through the inhibitory actions of SU9516 on the p65-NF-κB pro-inflammatory pathway and the Ste20-related proline alanine rich kinase (SPAK)/OSR1 signaling pathway.

## A small molecule integrin-enhancing compound for the treatment of DMD

This study was the first to demonstrate the potential of an α7β1 integrin enhancing drug as a therapeutic for DMD. Prior to our study, the compound SU9516, a known cyclin dependent kinase (cdk) inhibitor^19^ was investigated within the realm of anti-cancer therapeutics. Previously published literature on SU9516 sheds light on its ability to reduce cell proliferation, increase apoptosis and induce mitochondrial injury in various cancer cell lines^20,21^. However, due to the apparent non-specific inhibitory action of SU9516 on various other kinase pathways, the drug did not progress towards clinical trials as an anti-cancer therapeutic.

We showed that SU9516 increases levels of the α7β1 integrin complex in human DMD patient myotubes as well as *mdx* mice. In order to isolate the specific kinase pathway that was perhaps responsible for the increase in α7β1 integrin, we performed a biochemical KiNativ assay to identify kinase targets of SU9516, in human DMD patient myotubes. Among compounds evaluated in the initial drug screens were several cdk inhibitors with different selectivities that showed no increased expression of α7 integrin. Hence, we excluded cdks as possible therapeutic targets. We were surprised to find that in myotubes, the SPAK/OSR1 kinases were inhibited across all concentrations of SU9516 with an ~80% inhibition seen at the lowest concentration of 0.1 μM. By utilizing a known inhibitor of this pathway, we showed increased α7 integrin levels in myotubes, and thus demonstrated that blocking SPAK/OSR1 at least partly contributes to the increase in α7 integrin. However, further investigation is needed to understand whether the increase in integrin is dependent on a single pathway or additional pathways. Abolishing the SPAK, OSR1 or both kinases’ activities will help us to better understand the association between the inhibition of these kinases and integrin expression.

**Figure 1:**
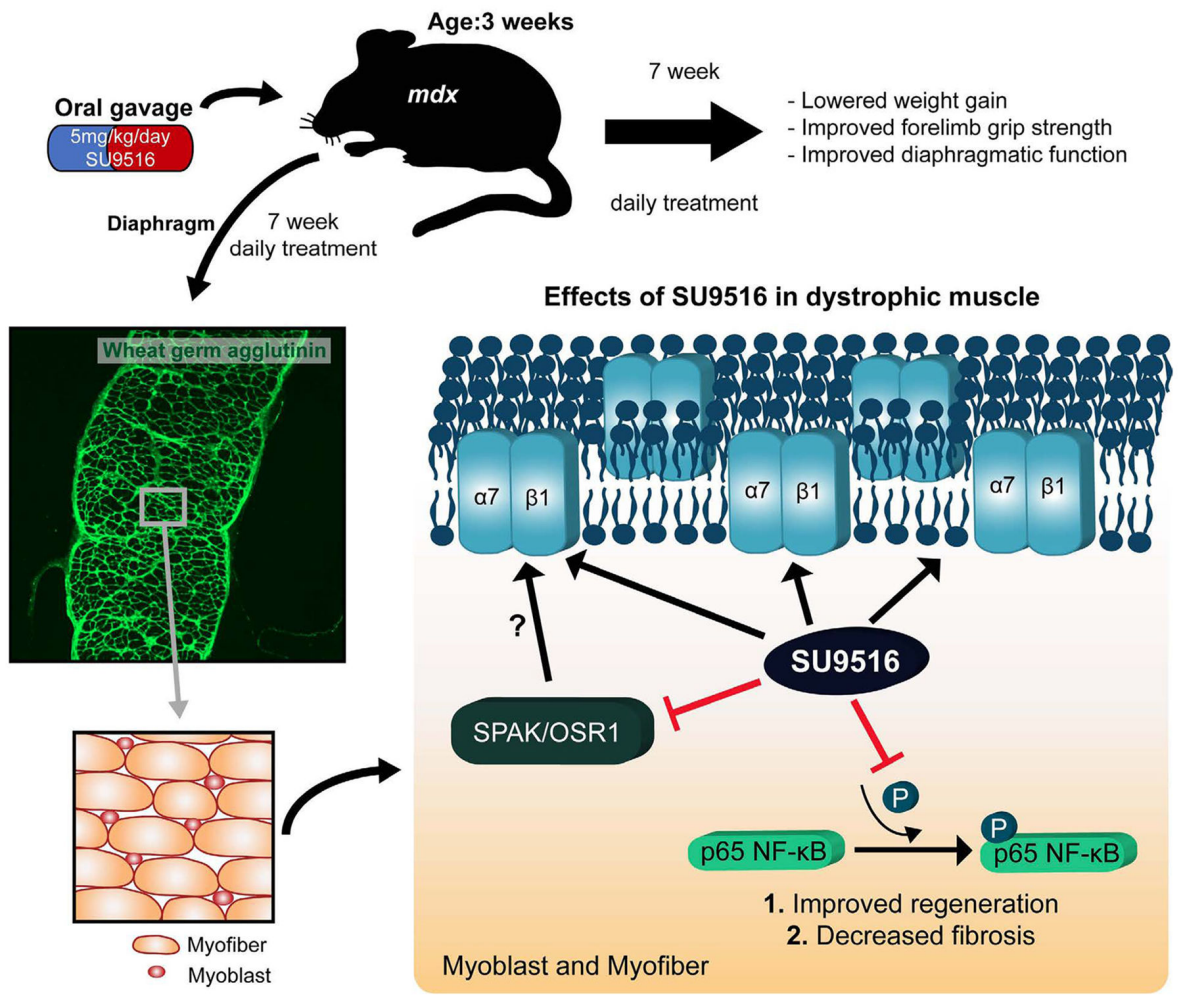
**Top panel:** The functional effects of a 7-week daily treatment regimen of orally administered SU9516 in the *mdx* mouse model of Duchenne muscular dystrophy**. Bottom panel:** Schematic depicting signaling effects of a 7-week daily treatment regimen of SU9516 in the inhibition of SPAK/OSR1 and the pro-inflammatory NF-κB pathway.

Following *in vitro* validation, preclinical studies were initiated where *mdx* mice were administered a daily dose of 5mg/kg SU9516 via oral gavage from 3 to 10 weeks of age. This dosing regimen resulted in significant improvements in body weight over the course of treatment. *Mdx* mice tend to gain more weight over time compared to their wild type counterparts^22^, and SU9516 treatment showed reduction in weight gain compared to vehicle-treated *mdx* mice. Additionally, forelimb grip strength measurements were significantly improved with SU9516 treatments. DMD patients suffer from severe diaphragmatic weakness resulting in respiratory dysfunction. Although the *mdx* mouse does not accurately depict the severe progression of the DMD disease phenotype in humans, the *mdx* diaphragm muscle shows severe functional deficits, damaged fibers, fibrosis and centrally nucleated fibers^23^. SU9516 treatment improved specific force defined as the force normalized to cross sectional area (CSA) [CSA (mm^2^) =mass (mg)/[(L_0_ mm) *(L/L_0_) *(1.06 mg/mm^3^)], where L/L_0_(fiber to muscle length ratio)=1 in the diaphragm, the value 1.06 is the density of muscle)] developed in the *mdx* diaphragm as evaluated using *ex vivo* experiments post completion of treatment course. Furthermore, SU9516 treatment promoted restoration of function post fatigue in the diaphragm. Accompanying the functional improvements, we detected an increase in the percentage of regenerating myofibers as evidenced by immunostaining for embryonic myosin heavy chain. To understand the mechanism by which an increase in regenerating myofibers was observed with SU9516, we looked at the p65 NF-κB pathway via immunoblotting to see if SU9516 inhibits this inflammatory pathway. We found a reduction in the levels of phosphorylated p65 NF-κB with SU9516 treatment in both human DMD myotubes as well as *mdx* mice. Previous reports have demonstrated that muscle derived stem cells from a haploinsufficient mouse model for p65 NF-κB exhibited enhanced myogenic differentiation^24^ which are the effects we observe with SU9516 treatment *in vitro* and *in vivo.* Additionally, SU9516 mediated inhibition of p65 NF-κB could partially explain the reduction in fibrosis as evidenced by Sirius Red staining in diaphragm cross sections. The SU9516 treatment paradigm and its beneficial effects in *mdx* mice through various mechanisms are summarized in Figure 1.

The results published in this study bring to light the efficacy of SU9516 in the treatment of DMD. A seven week, daily oral administration of SU9516 in *mdx* mice achieves therapeutic levels of α7β1 integrin in muscle, in keeping with integrin α7 overexpression transgenic studies in *mdx* mice13. However, there are still critical aspects of this study that must be addressed such as whether SU9516 depletes the satellite cell niche *in vivo*, while promoting myofiber regeneration in dystrophic muscle. Although our study adopted a daily oral administration regimen owing to the short half-life of the drug *in vivo*, the timing and drug concentrations are aspects of this study that must be carefully elucidated in preclinical studies. Additionally, it is unknown for how long the beneficial effects of the drug are sustained post suspension of drug dosing and it will be important to evaluate the long-term benefits of this compound even after its suspension. An important note mentioned in our original article is the fact that SU9516 is toxic in mice when administered via oral gavage at a concentration over 5 mg/kg. SU9516 was initially identified as a pro-apoptotic compound in cancer cell lines and this is an undesired property in a therapeutic for DMD, a disease characterized by necrotic death of myofibers. These side effects make it unlikely that SU9516 will be the molecule that will ultimately be administered to DMD patients. Modulation of chemistry or analogs of SU9516 will have to be investigated in preclinical models of muscular dystrophy to improve drug half-life and eliminate toxic side effects for clinical translation. Nevertheless, this study leads the way for further identification of other integrin enhancing compounds as well as the development of SU9516 analogs for progression towards clinical trials.
